# Data for a direct fibrinolytic metalloproteinase, barnettlysin-I from *Bothrops barnetti* (barnett^,^s pitviper) snake venom with anti-thrombotic effect

**DOI:** 10.1016/j.dib.2016.04.054

**Published:** 2016-04-30

**Authors:** Eladio Flores Sanchez, Michael Richardson, Luiza Helena Gremski, Silvio Sanches Veiga, Armando Yarleque, Stephan Niland, Augusto Martins Lima, Maria Inácia Estevao-Costa, Johannes Andreas Eble

**Affiliations:** a Research and Development Center, Ezequiel Dias Foundation, 30510-010 Belo Horizonte, MG, Brazil; b Department of Cell Biology, Federal University of Parana, Brazil; c Faculty of Biological Sciences, Nacional University of San Marcos, Lima, Peru; d Institute for Physiological Chemistry and Pathobiochemistry, University of Münster, Germany

## Abstract

Initial association of platelets after vascular injury is mediated by glycoprotein (GP)Ib-IX-V binding to von Willebrand factor (vWf) immobilized on exposed collagens and eventually leads to thrombus formation. This article provides data about a new P-I class snake venom metalloproteinase (SVMP), barnettlysin-I (Bar-I), purified from the venom of *Bothrops barnetti*. This Data in Brief manuscript complements the main research article by providing additional data of the biochemical characterization of Bar-I 10.1016/j.bbagen.2015.12.021[1].

**Specifications table**

**Table:** 

Subject area	*Biochemistry*
More specific subject area	*Toxicology of animal venoms: Protein purification from snake venom*
Type of data	*Figures*
How data was acquired	*in vitro assays for structure- function relationships*
Data format	*Analyzed*
Experimental factors	*Proteinase was purified by different chromatography steps*
Experimental features	*SDS-PAGE analysis, in vitro proteolytic activity assays, western blot*
Data source location	*B. barnetti venom was purchased from National Institute of Health, Lima-Peru.*
Data accessibility	*The data are supplied with this article*

## 1 Value of the data

– Data presented a new metalloproteinase barnettlysin-I (Bar-I) which directly degrades fibrin, a plasma protein that provides the scaffolding for blood clots.
– The enzyme has bifunctional activity and may represent a significant alternative over currently available antiplatelet drugs.
– This data could be of interest to researchers studying new and emerging thrombolytic and anti-thrombotic agents.

## 2 Data

**Fig. 1:**
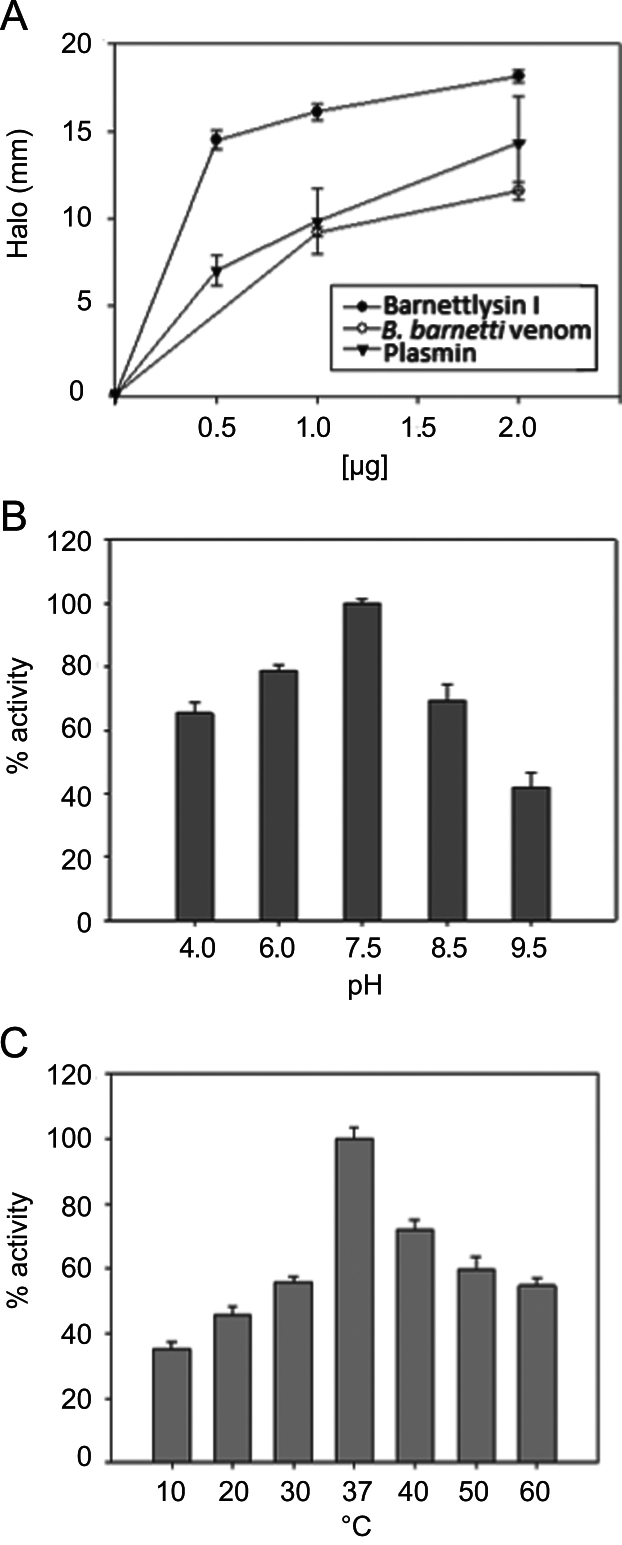
Measurement of proteolytic activity of Bar-I. (A) Fibrinolytic activity of Bar-I. The fibrinolytic activity was measured in triplicates by the halo size on a fibrin bed following overnight incubation at 37 °C with several concentrations of Bar-I, crude venom of *B. barnetti* and plasmin (0.5–2.0 μg/μl). The data are expressed as means of triplicate assays with standard errors. Effect of pH (B) and temperature (C) on the proteolytic activity of Bar-I using DMC as substrate. Each point represent the mean and standard error of three individual experiments.

**Fig. 2:**
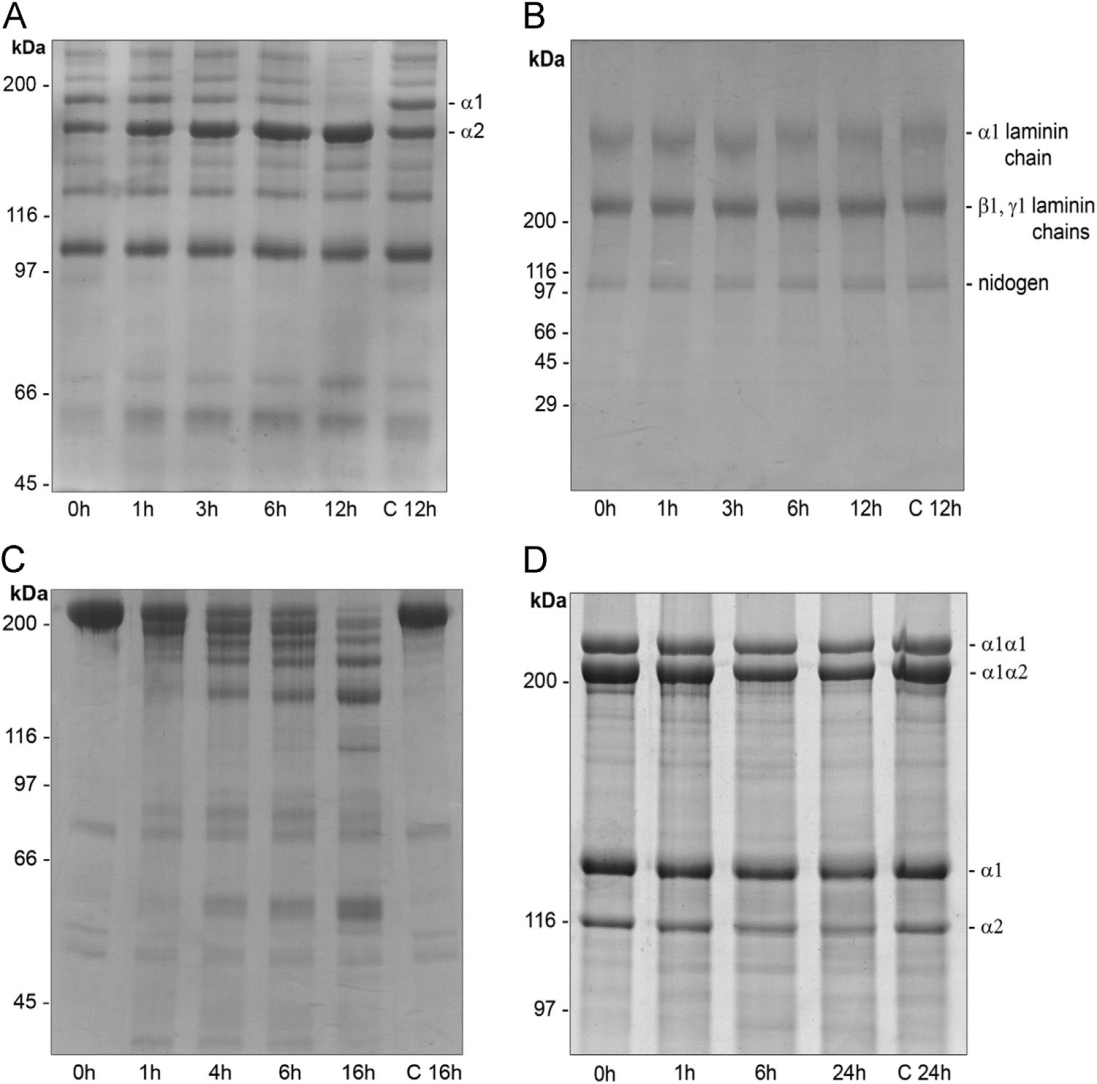
Proteolytic degradation of collagens type IV and I, laminin/nidogen and fibronectin by Bar-I. (A) SDS-PAGE (7.5%) analysis of collagen type IV after incubation with Bar-I for 1, 3, 6 and 12 h at 25 °C at rate of 1:50 (w/w). Type IV collagen chains α1 and α2 are indicated (B) SDS-PAGE (3-20% gradient) of laminin and nidogen after incubation with Bar-I for 1, 3, 6 and 12 h at 37 °C at rate of 1:50 (enzyme:substrate). Typical laminin chains (α1, β1 and γ1) are indicated. (C) Purified human plasma fibronectin samples were incubated with Bar-I for 1, 4, 6 and 16 h at 37 °C at rate of 1:100 (enzyme:substrate), aliquots of the incubation mixtures were analyzed by SDS-PAGE (7.5%). (D) SDS-PAGE (5%) analysis of collagen I after incubation with Bar-I for 1, 6 and 24 h at 37 °C at rate of 1:50 (enzyme:substrate). Typical type I collagen chains (α1α1, α1α2, α1 and α2) are indicated. Position of the molecular mass markers are indicated on the left of the gels. Lane C depicts control molecules (without any Bar-I treatment).

**Fig. 3:**
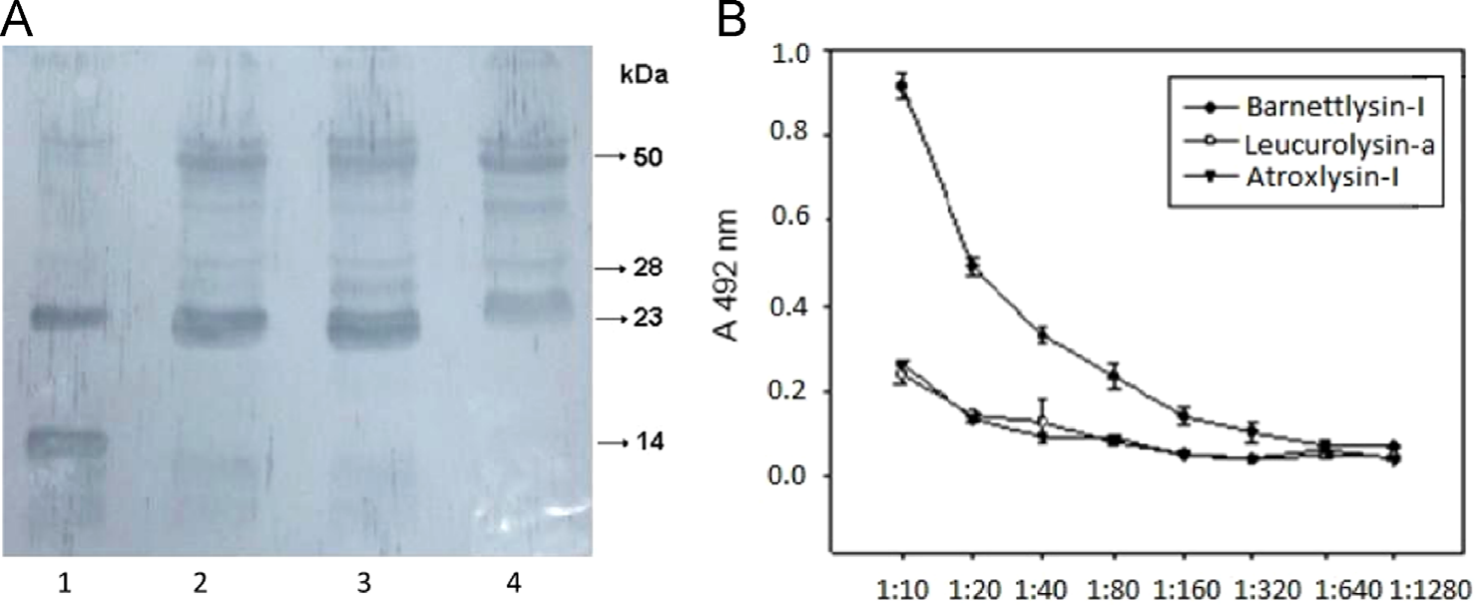
Reactivity of anti-Bar-I IgG against Bothrops venoms and P-I SVMPs analyzed by western blot and indirect ELISA. (A) Immunobloting of anti-Bar-I IgG against Bar-I (5 μg, lane 1) and venoms (15 μg each) of *B. barnetti* (lane 2), *B. atrox* (lane 3) and *B. pictus* (lane 4). (B) 96-well microtitration plates were precoated with 0.5 μg/well of purified barnettlysin-I (-●-), leucurolysin-a (-o-) and atroxlysin-I (-▼-). Anti-Bar-I IgG was added at different dilutions. Binding was visualized by incubation with peroxidase-coupled anti-rabbit IgG (diluted 1:12000) and by further addition of o-phenylenediamine. The absorbance of pre-immune serum was substracted. These values are means of three independent experiments.

This dataset describes some conditions that affect Bar-I proteolytic activity which was measured with fibrin and dimethylcasein (DMC) as substrates. Furthermore, the effect of the venom enzyme using the major base membrane (BM) components and human body, collagens IV, I, laminin-enactin/nidogen complexes, and plasma fibronectin (FN) was evaluated and analyzed by SDS-PAGE. In addition, immunological cross-reactivity of the purified rabbit anti-Bar-I IgG with related proteins bands from the venoms of *B. barnetti*, *B. atrox* and *B. pictus,* was assessed by western blotting and quantified by ELISA. Fig. 1, Fig. 2, Fig. 3

## 3 Experimental design, materials and methods

### 3.1 Enzyme activity of barnettlysin-I

The enzymatic activity of Bar-I was measured by DMC assay as described [2] and by the fibrin plate lysis method [3]. For comparative purposes, crude venom and plasmin were included in the fibrinolytic assays. To evaluate the effects of pH and temperature on proteolytic activity of Bar-I, DMC was used as substrate. The pH activity profile was obtained by incubating Bar-I (1 µg) in the following buffers for 2 h at room temperature: 20 mM citrate buffer (pH 4.0), 20 mM Hepes (pH 6.0–7.5), and 20 mM Tris–HCl (pH 8.5–9.5), all buffers containing 0.5 mM CaCl_2_ and 10 mM NaCl. The thermal stability of Bar-I was examined by heating the sample (1 µg) in 50 mM Hepes buffer, pH 7.5, containing 0.5 mM CaCl_2_ for 15 min. After this time, the samples were cooled in an ice bath and the proteinase activity was measured on DMC.

### 3.2 Proteolytic activity upon plasma fibronectin (FN) and some extra cellular matrix (ECM) proteins

The activity of Bar-I on plasma FN and some ECM proteins was determined by incubating laminin, type IV and I collagens at the enzyme:substrate ratio of 1:50 (w/w) and FN at the ratio of 1:100 (w/w) in 50 mM Hepes buffer, pH 7.5 for different time intervals at 37 °C. Digestion products were analyzed by SDS-PAGE.

### 3.3 Immunoblot and enzyme-linked immunoabsorbent assay (ELISA) analysis

Samples for immunoblot analysis were subjected to reduced SDS-PAGE (12% gel), and the proteins were transferred onto nitrocellulose membranes using the Bio-Rad transblot-apparatus according to the manufacturer׳s instructions. Blotting procedures, using purified rabbit IgG (4 µg) against Bar-I were performed. For ELISA, microtiter plates were coated overnight with 0.5 µg/well of each P-I enzymes (Bar-I, leucurolysin-a and atroxlysin-I) in 0.05 M carbonate buffer, pH 9.6 (100 µl, standard volume). After washing with 0.05% Tween saline, a blocking solution (2% casein in phosphate buffered saline, PBS) was added (1 h at room temperature). After two additional washing steps with the same solution, anti-Bar-I IgG diluted in PBS containing 0.25% casein and 0.05% Tween 20 was incubated for 1 h at 37 °C. After six washes, peroxidase-coupled anti-rabbit antibody (Sigma, diluted 1:12000) was added for 1 h at room temperature. The wells were washed and 100 µl of peroxidase substrate o-phenylenediamine (0.33 mg/ml in citrate buffer, pH 5.2 in the presence of 0.04% hydrogen peroxide) was added and the color reaction developed for 1 h at 37 °C in a dark. Absorbance was read with a micro-plate reader at 492 nm. All measurements were made in triplicate.
