# Supplementary figures and images for: Data for a direct fibrinolytic metalloproteinase, barnettlysin-I from Bothrops barnetti (barnett,s pitviper) snake venom with anti-thrombotic effect
Source: Data Brief. 2016 Apr 30;7:1609–13. doi: 10.1016/j.dib.2016.04.054 (PMC4865631; doi:10.1016/j.dib.2016.04.054)

Bar-I, Supplementary Figs 1, 2 and 3

**A**

Halo (mm)


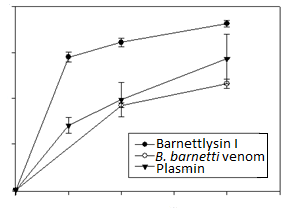


0.5 1.0 1.5 2.0

20

15

10

5

0

**B**

[µg]

**B**


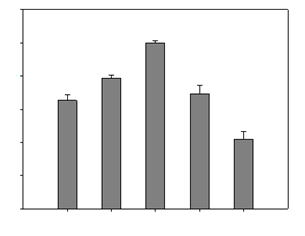


120

100

80

60

40

20

4.0 6.0 7.5 8.5 9.5

pH

0

% activity

**C**


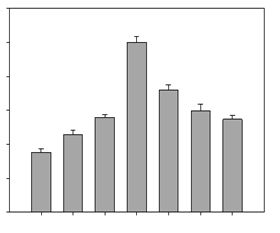


10 20 30 37 40 50 60

°C

0

20

40

60

80

100

120

% activity

Figure 1 -





Figure 2 -

**B**

**A**


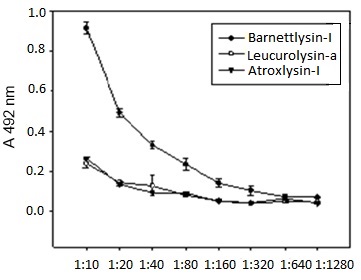


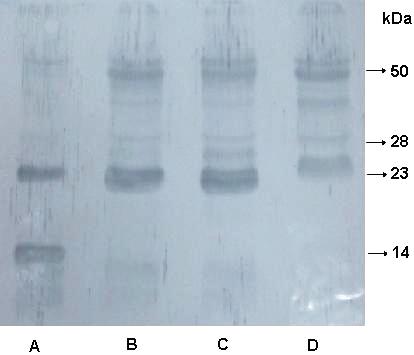


1 2 3 4

Figure 3 -

Supplement: Supplementary file 1 — Supplementary material [file mmc1.zip › Bar-I suplementary material.docx]
