# Supplementary material for: Data for a direct fibrinolytic metalloproteinase, barnettlysin-I from Bothrops barnetti (barnett,s pitviper) snake venom with anti-thrombotic effect
Source: Data Brief. 2016 Apr 30;7:1609–13. doi: 10.1016/j.dib.2016.04.054 (PMC4865631; doi:10.1016/j.dib.2016.04.054)
Supplement: Supplementary file 2 — Supplementary material [file mmc2.docx]

**Conflict of interest:** The authors declare no competing financial interests.
